# Overexpression of Retinal Degeneration Slow (RDS) Protein Adversely Affects Rods in the *rd7* Model of Enhanced S-Cone Syndrome

**DOI:** 10.1371/journal.pone.0063321

**Published:** 2013-05-01

**Authors:** Dibyendu Chakraborty, Shannon M. Conley, Muna I. Naash

**Affiliations:** Department of Cell Biology, University of Oklahoma Health Sciences Center, Oklahoma City, Oklahoma, United States of America; Cedars-Sinai Medical Center, United States of America

## Abstract

The nuclear receptor NR2E3 promotes expression of rod photoreceptor genes while repressing cone genes. Mice lacking NR2E3 (*Nr2e3^rd7/rd7^* referred to here as *rd7*) are a model for enhanced S-cone syndrome, a disease associated with increased sensitivity to blue light and night blindness. *Rd7* retinas have reduced levels of the outer segment (OS) structural protein retinal degeneration slow (RDS). We test the hypothesis that increasing RDS levels would improve the *Rd7* phenotype. Transgenic mice over-expressing normal mouse peripherin/RDS (NMP) in rods and cones were crossed onto the *rd7* background. Disease phenotypes were assessed in NMP/*rd7* eyes and compared to wild-type (WT) and *rd7* eyes at postnatal day 30. NMP/*rd7* retinas expressed total RDS (transgenic and endogenous) message at WT levels, and NMP protein was correctly localized to the OS. NMP/*rd7* retinas have shorter OSs compared to *rd7* and WT and significantly reduced number of rosettes. NMP/*rd7* mice also exhibited significant deficits in scotopic ERG amplitudes compared to *rd7* while photopic amplitudes remained unaffected. Protein levels of rhodopsin, RDS, and the RDS homologue ROM-1 were significantly reduced in the NMP/*rd7* retinas compared to *rd7*. We show that correcting the levels of RDS gene expression does not improve the phenotype of the *rd7* suggesting that RDS deficiency is not responsible for the defect in this model. We suggest that the specific rod defect in the NMP/*rd7* is likely associated with ongoing problems in the *rd7* that are related to the expression of cone genes in rod cells, a characteristic of the model.

## Introduction

Retinal development and photoreceptor differentiation is guided by a precisely regulated series of transcription factors. The homeobox genes CRX and OTX regulate both rod and cone genesis. Downstream of these are NRL and NR2E3 (also called PNR) [1]. NRL is required for developing cells to adopt a rod fate; in the *Nrl^−/−^* mouse model, rods fail to form and instead become S-cone like [2]. NR2E3 is a direct downstream target of NRL [3] and functions as a co-activator of rod genes as well as a suppresser of cone genes [4], [5], [6]. Mutations in the *NR2E3* gene are associated with enhanced S-cone syndrome (ESCS) in patients [7] which manifests as increased sensitivity to blue light (mediated by S-cones), and night blindness (due to rod defects). Other phenotypes associated with ESCS can include some loss of L- and M-cone vision, as well as retinal tearing and retinal neovascularization [7], [8], [9]. Mutations within *NR2E3* are also associated with other retinal diseases, including Goldman-Favre syndrome, clumped pigmentary retinopathy, and autosomal dominant retinitis pigmentosa (ADRP) [10], [11], [12], [13], [14].

The retinal degeneration 7 (*Nr2e3^rd7/rd7^* here referred to as *rd7* for the sake of simplicity) mutant mouse lacks NR2E3 and has been used as a model for ESCS [15], [16]. This mouse exhibits an approximately 2-fold increase in blue opsin (S-opsin) expressing cells [16], [17]. This increase in S-cones was originally attributed to abnormal proliferation, but recent data suggests that these S-cones are actually arise from a small population of early-born rod progenitors [18]. *Rd7* mice exhibit rosettes in the outer nuclear layer (ONL), slow retinal degeneration, and abnormal electroretinograms (ERG) [15]. Specifically, they show decreased rod ERG amplitude, and no increase in cone ERG response in spite of the increase in cone number [15]. In addition to these characteristics, the *rd7* eyes demonstrate substantial alterations in the pattern of retinal gene expression, including de-repression of many cone-specific genes [5], [17].

One of the transcriptional targets of NR2E3 is the photoreceptor-specific protein retinal degeneration slow (RDS). In the *rd7* retinas, the levels of RDS message are reduced by ∼3.2 fold compared to wild-type (WT) [19]. RDS is localized to the rim region of rod and cone outer segment (OS) discs/lamellae and is required for proper photoreceptor OS morphogenesis and structure [20], [21]. Mutations in the RDS gene are associated with a variety of inherited human retinal diseases, including ADRP and multiple classes of macular degeneration [22], [23]. The level of RDS expression has shown to be critical to OS structure and function. RDS haploinsufficiency (for example in the *rds^+/−^* mouse) results in an ADRP-like phenotype, characterized by 1) early defects in rod function and later onset defects in cone function, 2) shortened OSs with membranous whorls, and 3) slow degeneration. Similarities between some RDS mutant mouse models [19] and the course of degeneration in the *rd7* combined with the decreased levels of RDS expression in the *rd7*, have led to the hypothesis that part of the degenerative phenotype in the *rd7* may be due to RDS haploinsufficiency [19].

To test this hypothesis, we took advantage of a transgenic mouse model we have generated to over-express wild-type RDS (normal mouse peripherin/RDS [NMP]). This transgene is driven by the interphotoreceptor retinoid binding protein (IRBP) promoter and previous characterization has established that one NMP allele results in transgenic RDS protein levels which are ∼30% of WT levels [24]. Our work using this model has shown that there are no detrimental effects of RDS overexpression (i.e. NMP on a WT background) on the functional, structural, or biochemical level, and that this transgene can mediate structural and functional improvement in the *rds^+/−^* haploinsufficiency model of ADRP [24] and in the C214S-RDS transgenic model of ADRP [25]. Here, we bred the NMP transgene onto the *rd7* background and evaluated the retinal phenotype. Surprisingly, instead of mediating improvement in the *rd7* phenotype, over-expression of RDS exacerbated the degenerative phenotype.

## Results

### Expression and localization of NMP in the *rd7* retina

**Figure 1:**
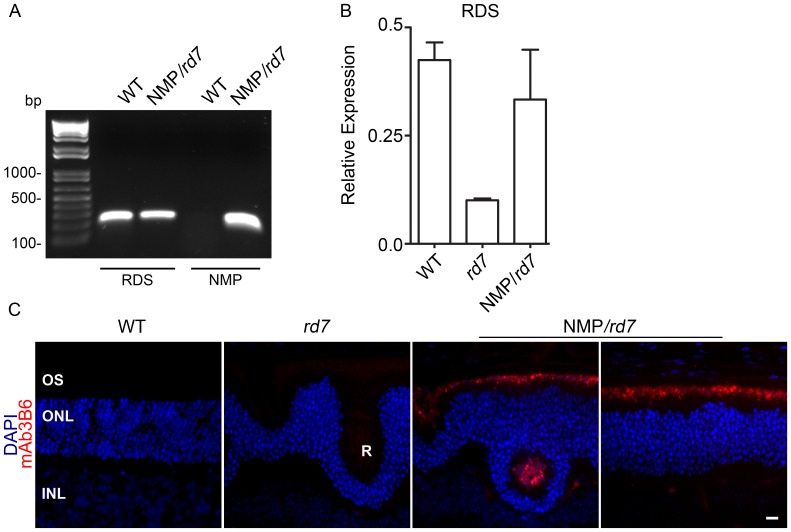
The NMP transgene is properly expressed in the *rd7* retina. **A.** RT-PCR was used to detect the presence of the NMP transgene at P30. **B.** qRT-PCR was used to measure the levels of total RDS also at P30 in eyes of the indicated genotypes. Data shown here are means±SEM from 3 different eyes/group. **C.** Paraffin-embedded retinal sections were labeled with mAb 3B6 (red) which specifically recognizes NMP protein but not endogenous RDS. OS: outer segment, ONL: outer nuclear layer, INL: inner nuclear layer, R: rosette. Scale bar, 10 µm.

To test the hypothesis that increasing RDS levels in *rd7* retinas could ameliorate the degenerative phenotype, we crossed the NMP transgenic line onto the *rd7* background. We generated retinal cDNA and amplified using primers that recognize both endogenous RDS and NMP (**Table S1**, and **Fig. 1A**, first two lanes) as well as primers that are specific for the NMP transgene (**Table S1**, and **Fig. 1A**, second two lanes). Amplicons were the predicted size and confirm that the NMP transgene is expressed. We next undertook qRT-PCR in the three models at postnatal day (P) 30 using a primer set that amplifies from endogenous and transgenic message. Consistent with previous studies, RDS message levels are significantly reduced in the *rd7* (**Fig. 1B**). Expression of the NMP transgene (NMP/*rd7*) brought total RDS message levels back to WT levels (**Fig. 1B**). The NMP transgene contains a C-terminal modification (P341Q) which does not affect the function of NMP protein [24], but enables specific recognition of the NMP protein in the presence of endogenous RDS using the monoclonal antibody mAb 3B6. Immunohistochemistry with this antibody demonstrated that the NMP protein is properly localized to the OSs (**Fig. 1C**) in the NMP/*rd7*. No signal was observed in WT or *rd7* retinas, consistent with our prior observations on the specificity of mAB 3B6 [24], [26].

### Overexpression of RDS adversely affects OS structure in the *rd7* background

**Figure 2:**
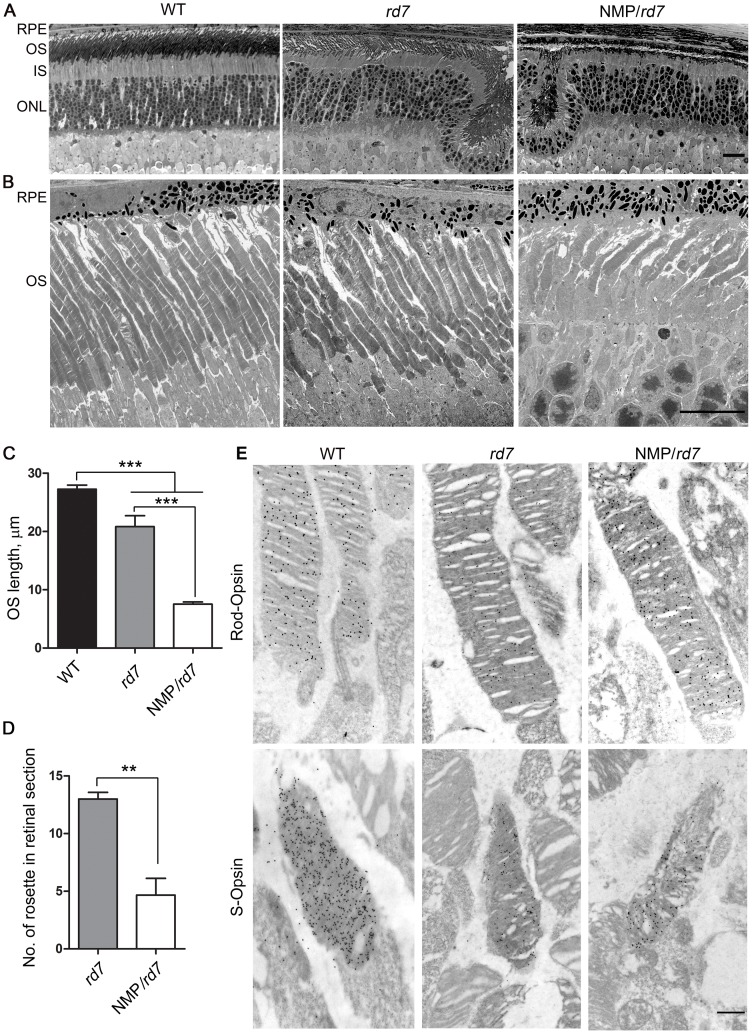
Over expression of RDS causes OS segment degeneration. Shown are representative light (**A**) and electron micrographs (**B**) from retinal section of WT, *rd7* and NMP/*rd7* at P30. **C**. OS length in images captured from the central retina was measured in 3 fields/eye and averaged from 3–4 different eyes/genotype at P30. *** P<0.001 by one-way ANOVA with Bonferroni's post-hoc comparisons. **D**. The number of rosettes in 3 sections/eye was counted along the inferior/superior plane and averaged to give a per eye value. N = 3 different eyes/genotype. ** P<0.01 by Student's t-test. **E**. Immunogold labeling of rod and cone OSs at P30 using rod-opsin and S-opsin antibodies. RPE: retinal pigment epithelium, OSL outer segment, IS: inner segment, ONL: outer nuclear layer. Scale bar 20 µm (**A**), 10 µm (**B**), 500 nm (**E**).

We examined the overall structure of the retina and OSs in WT, *rd7*, and NMP/*rd7* mice at P30 (**Fig. 2A&B**). As previously reported [19], the OSs in *rd7* are moderately reduced in length; here we find a 24% reduction compared to WT (**Fig. 2C**). Strikingly, this reduction in OS length is significantly increased in NMP/*rd7* (76% reduction compared to WT). This difference can also be seen on the ultrastructural level; while most rods in the NMP/*rd7* still contain nicely stacked discs, they are much shorter than WT or *rd7* rods (**Fig. 2B**). These shortened OSs significantly differ from the *rds^+/−^*, which are characterized by whorls of membrane [27], [28] and do not exhibit stacked discs.

In common with some other models of retinal degeneration, the *rd7* exhibits rosettes in the ONL (**Fig. 2A**). These rosettes begin to attenuate by 5 months of age and disappear by 16 months of age [15]. The *Nrl^−/−^* retina also exhibits rosettes which disappear as the retina degenerates, and we have observe that this disappearance is accelerated by the presence of additional degenerative mutations [29], [30]. We quantified the number of rosettes in central retinal sections from *rd7* and NMP/*rd7*, and show that there is a significant reduction in the number of rosettes found in the NMP/*rd7* compared to the *rd7* at P30 (**Fig. 2D**) suggesting that the presence of the NMP transgene accelerates the rate of degeneration in the *rd7*. EM/immunogold labeling with antibodies against short wavelength cone opsin (S-opsin) to label cone OSs (**Fig. 2E**) did not reveal any obvious ultrastructural changes between cones in the three models, although quantitative morphometry was not feasible due to the small number of cones.

### Levels of OS proteins are significantly decreased in the NMP/*rd7* retina

**Figure 3:**
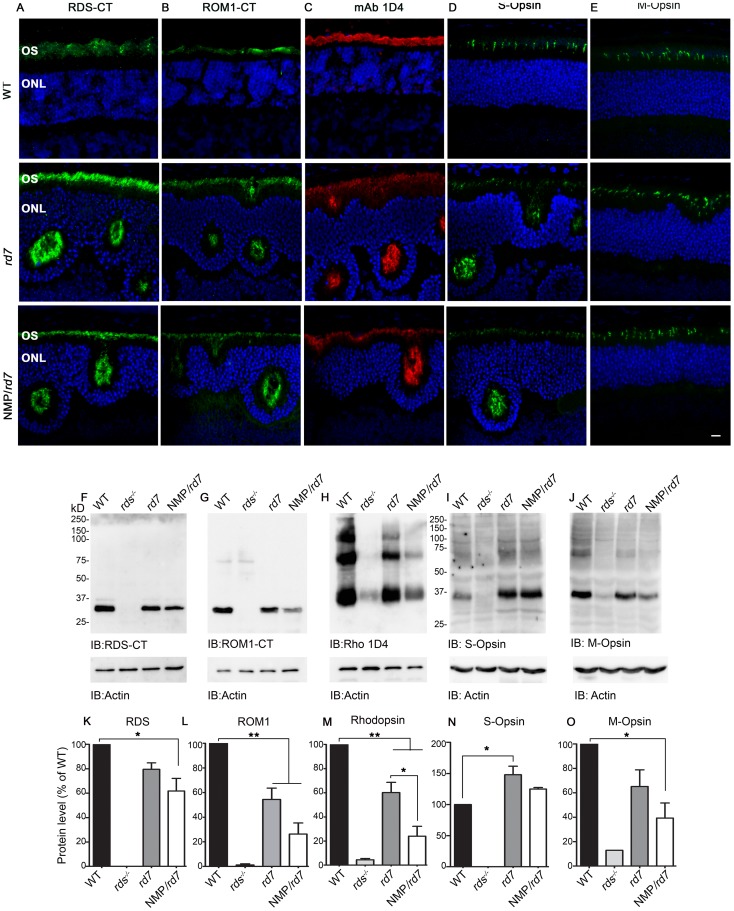
Expression of OS proteins is decreased in the NMP/*rd7*. Paraffin embedded retinal sections from WT, *rd7* and NMP/*rd7* were labeled with (**A**) anti RDS-CT, (**B**) anti-ROM1-CT, (**C**) mAB 1D4 against rhodopsin, (**D**) anti-S-opsin, and (**E**) anti-M-opsin, and were all counterstained with DAPI. Retinal extracts were isolated from P30 WT, *rds^−/−^* (negative control), *rd7*, and NMP/*rd7* and were analyzed by reducing SDS-PAGE/western blot. The blots were probed with (**F**) anti RDS-CT, (**G**) anti-ROM1-CT, (**H**) mAB 1D4 against rhodopsin, (**I**) anti-S-opsin, and (**J**) anti-M-opsin antibodies. Blots were also labeled with actin-HRP as a loading control. Protein was quantified densitometrically and normalized to actin. Levels of OS proteins were measured in 3–6 retinas per genotype: total RDS (**K**), ROM-1 (**L**), rhodopsin (**M**), S-opsin (**N**), and M-opsin (**O**). Data are presented means±SEM from 5–6 different retinas/genotype. *P<0.05, **P<0.01, by 1-way ANOVA with Bonferroni's post-hoc comparisons. OS: outer segment, ONL: outer nuclear layer. Scale bar 10 µm.

To more fully characterize the effects of the NMP transgene, we used immunohistochemistry (IHC) to assess subcellular localization of a panel of OS proteins at P30 (**Fig. 3A–E**). Because our IHC is not quantitative, we then used western blotting to assess levels of protein expression (**Fig. 3F–O**). Although some other models of retinal degeneration exhibit mislocalization of OS proteins as part of the degenerative process [31], [32], here we saw no mislocalization of transgenic RDS (**Fig. 1C**), total RDS (the RDS-CT antibody recognizes both transgenic and endogenous RDS, **Fig. 3A**), the RDS binding partner ROM-1 (**Fig. 3B**), rhodopsin (**Fig. 3C**) or S- and M-cone opsins (**Fig. 3D–E**). Levels of RDS protein were moderately reduced in the *rd7* compared to WT; however, in contrast to the RDS message levels shown in **Fig. 1B**, RDS protein levels in NMP/*rd7* eyes were significantly lower than levels in both WT and *rd7* (**Fig. 3F, K**). A similar trend was seen for the RDS binding partner ROM-1 (**Fig. 3G, L**) and for rhodopsin (**Fig. 3H, M**). As reported previously, S-opsin levels were up in *rd7* compared to WT, and were not significantly altered in the NMP/*rd7* (**Fig. 3I, N**). M-opsin levels were also reduced in both *rd7* and NMP/*rd7* (**Fig. 3J, O**). These results suggest that levels of rod and M-cone photoreceptor proteins are adversely affected in the NMP/*rd7*, an outcome consistent with the dramatic OS shortening shown in **Fig. 2C**.

### Expression of NMP leads to a negative effect on rod but not cone function

**Figure 4:**
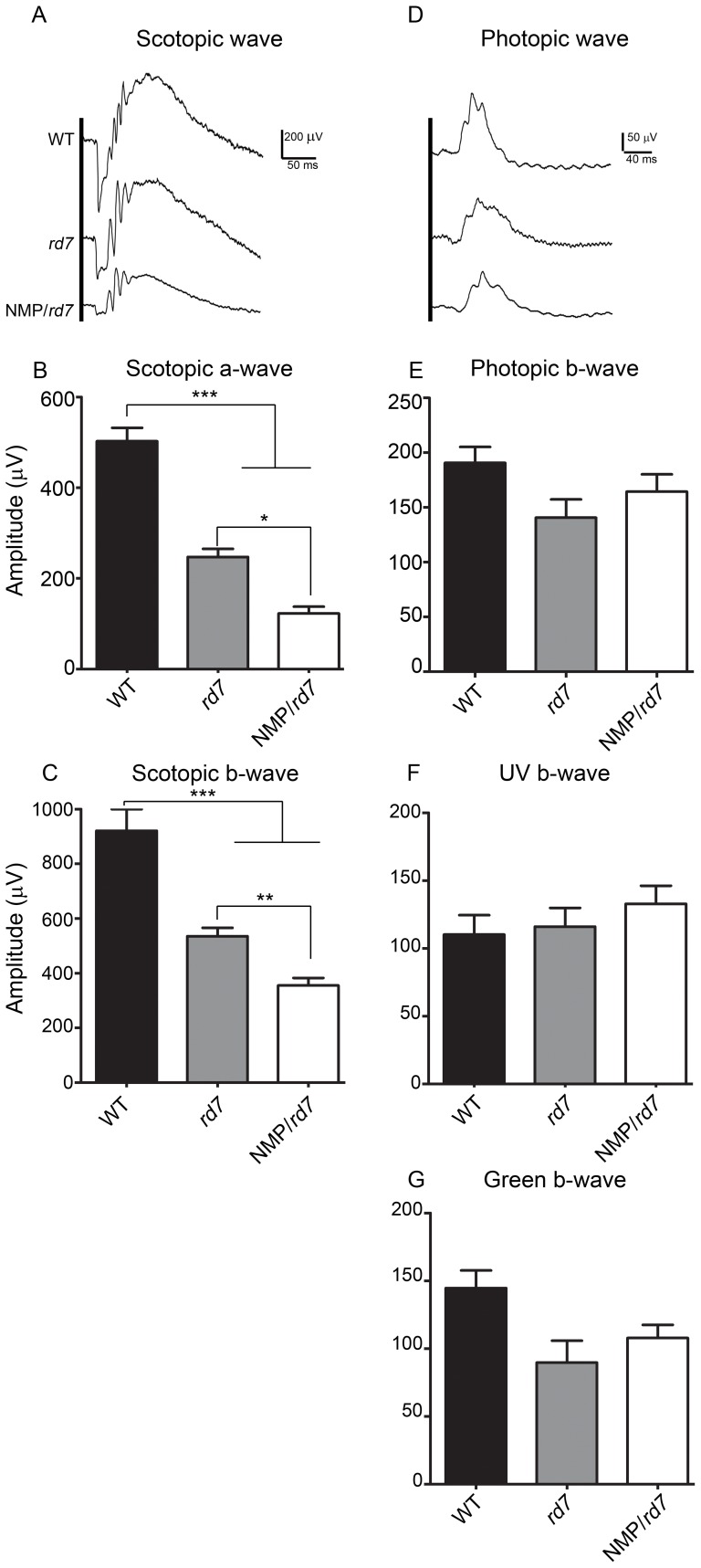
Expression of NMP in *rd7* leads to defects in rod function. Full-field scotopic (**A–C**) and photopic (**D–G**) were recorded at P30 from the indicated genotypes. **A** and **D** show representative scotopic and photopic wave forms. A significant reduction in maximum scotopic a-wave and b-wave amplitude was observed in NMP/*rd7* in comparison to WT and *rd7* (**B & C**). Photopic b-wave amplitudes in response to white (**E**), UV (**F**), and green (**G**) light were not different in any genotype. Data are presented means±SEM from 5–7 different mice/genotype, **P<0.01, ***P<0.001 by 1-way ANOVA with Bonferroni's post-hoc comparisons. N = 8 mice/genotype.

We next assessed retinal function at P30 by scotopic and photopic ERG (**Fig. 4**). Rod photoreceptor maximum amplitudes (ERG traces-**Fig. 4A**, a-wave amplitudes-**Fig. 4B**, b-wave amplitudes-**Fig. 4C**) were significantly reduced in *rd7* compared to WT, and were further reduced in NMP/*rd7* (compared to WT and *rd7*). In contrast, photopic b-waves in response to white (**Fig. 4D, E**), UV (S-cones, **Fig. 4F**), and green (M-cones **Fig. 4G**) light were not significantly different in the NMP*/rd7* compared to any other genotype. We, in common with others [15], do not see an increase in photopic b-wave amplitudes in the *rd7* compared to WT, even though S-opsin levels are significantly higher (see **Fig. 3**). Although the difference was not statistically significant, mean photopic b-wave amplitudes in response to green light tended to be lower in *rd7* and NMP/*rd7*, consistent with the modest reduction in M-opsin levels we observed (refer to **Fig. 3I, N**). These results show that a significant rod-targeted functional defect accompanies the structural and biochemical defects which occur in the NMP/*rd7* when compared to WT or *rd7*.

### RDS oligomerization is not altered in *rd7* or NMP/*rd7*

**Figure 5:**
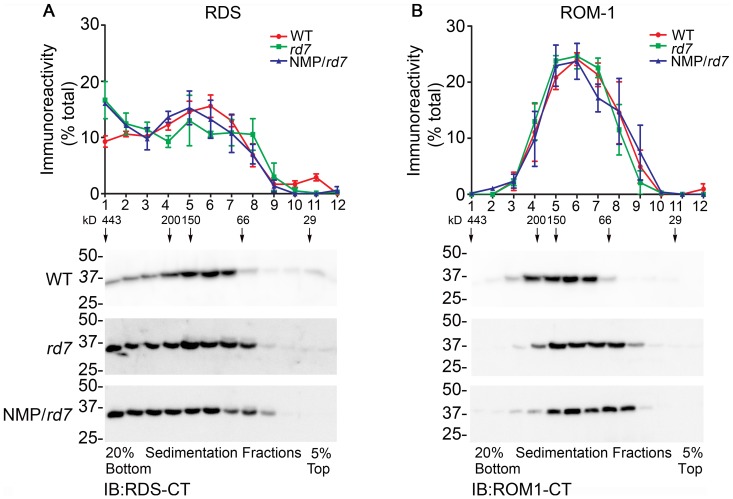
RDS complex formation is not altered in *rd7* or NMP/*rd7*. Non-reducing sucrose gradient velocity sedimentation on retinal extracts from WT, *rd7* and NMP/*rd7* was analyzed by reducing western blot (**A**, **B**, bottom). Blots were probed with anti-RDS-CT (**A**) and anti-ROM1-CT (**B**). Data are presented means±SEM from 3–4 independent experiments, each of which utilized retinas from separate animals. Graphs plot % of total RDS or ROM-1 found in each fraction.

Having thus described a severe rod-targeted degenerative phenotype when excess RDS is expressed in the *rd7* background, we wanted to understand the underlying cause of this defect. Proper OS rim formation requires that RDS assembles into a variety of different types of oligomeric complexes with its binding partner ROM-1. To understand whether excess RDS in *rd7* background results in alteration in RDS complex formation and whether these complexes are altered in the *rd7*, we undertook velocity sedimentation experiments (**Fig. 5**). Retinal extracts were fractionated on continuous, non-reducing 5%-20% sucrose gradients and then separated on reducing SDS-PAGE. Western blots were probed with antibodies against total RDS (RDS-CT) (**Fig. 5A**, bottom) and ROM-1 (ROM1-CT) (**Fig. 5B**, bottom). **Fig. 5A&B**, top show the percent of total RDS or ROM-1 in each fraction. In the WT retina, RDS is present as tetramers (fractions 6–9), octamers (fractions 4–5) and higher order oligomers (fractions 1–3), while ROM-1 is detected only as a tetramer and octamer. We do not observe any gross changes in RDS and ROM-1 complex formation in either the *rd7* or in the NMP/*rd7* compared to WT. These data suggest that interrupted RDS complex formation does not underlie the defect seen in the NMP/*rd7*. We do, however, observe a slight increase in the amount of RDS found in fractions associated with higher-order oligomers in both NMP/*rd7* and *rd7* extracts (fractions 1–2, green and blue lines compared to red in **Fig. 5A**). We have previously observed this phenomenon in the *Nrl^−/−^* model [29] and have hypothesized that a higher percent of RDS is found in higher-order oligomers in cones than in rods.

### Potential role of IRBP in retinal defects in the NMP/*rd7*

**Figure 6:**
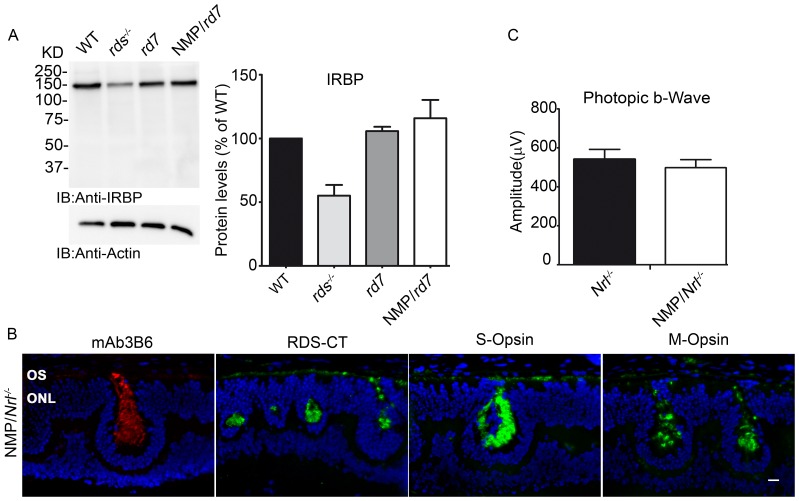
Changes in gene regulation *per se* do not underlie the defect in the NMP/*rd7*. **A**. Shown is a representative reducing SDS-PAGE/western blot probed with antibodies against IRBP. Densitometric quantification (normalized to actinis shown on the right (N = 3–4 retinas/genotype). **B–C**. The NMP transgene was crossed onto the *Nrl^−/−^* background. **B**. Localization of NMP (mAB 3B6), total RDS (RDS-CT), and cone opsins (S-opsin, M-opsin) was normal in the NMP/*Nrl^−/−^* at P30. **C**. Full-field photopic b-wave amplitudes in response to white light are not different in NMP/*Nrl* ^−/−^ compared to *Nrl* ^−/−^. Data are presented means±SEM from 8 mice/genotype *P<0.05, **P<0.01, ***P<0.001 by 1-way ANOVA with Bonferroni's post-hoc comparisons. for **A**, for **C**. OSs: outer segment, IS: inner segment, ONL: outer nuclear layer. Scale bar 10 µm.

IRBP expression is known to be regulated by NR2E3 during development (at P2), although not in the adult [33], and the NMP transgene is driven by the IRBP promoter. We hypothesized that the NMP transgene might sequester transcription factors needed for endogenous IRBP expression, and that altered IRBP levels in the NMP/*rd7* might account for part of the negative phenotype seen in these mice. However, western blots from retinas harvested at P30 show that IRBP levels are normal in both the *rd7* and NMP/*rd7* compared to WT (**Fig. 6A**).

### Expression of NMP on the *Nrl^−/−^* background does not cause retinal defects

Because the *Nrl^−/−^* model also exhibits altered retinal transcription, we next assessed the effects of the NMP transgene on the *Nrl^−/−^*. As in the WT (refer to **Fig. 3A**) and NMP/*rd7* (refer to **Fig. 1C**), in the NMP/*Nrl^−/−^* endogenous RDS, transgenic NMP (mAB 3B6), and both S- and M-opsin are properly localized (**Fig. 6B**). However, in stark contrast to the NMP/*rd7*, there is no functional defect in the NMP/*Nrl^−/−^* (**Fig. 6C**). These results suggest that the degenerative effects of the NMP transgene are due to some process specific to the *rd7*, and are not simply due to alterations in the retinal transcription networks.

## Discussion

Although overexpression of rhodopsin leads to retinal degeneration [34], [35], our previous study showed that overexpression of RDS in wild-type retinas does not cause any negative effects and can improve OS structure and function in the *rds^+/−^* and *rds^−/−^* backgrounds [24]. We hypothesized that if we corrected the RDS deficiency which occurs in the *rd7* using the NMP transgene, we might be able to improve the *rd7* phenotype. However, our results here show that the opposite is the case; expression of NMP in *rd7* retinas leads to a striking structural, functional, and biochemical defect in rods, with accompanying accelerated degeneration as indicated by a decrease in the number of rosettes.

One of the interesting outcomes we noticed here is that in both the *rd7* and the NMP/*rd7* we observe a small increase in the percent of RDS found in higher order oligomeric fractions. We have previously reported this phenomenon in the *Nrl^−/−^* background [29], [36]. Although the biological significance and mechanism underlying this difference are not known, it may contribute to the well-established differential role of RDS in rods vs. cones [30], [37], [38]. Why such a difference in the relative quantity of RDS higher-order oligomers should occur in the *rd7*, however, is not clear. The small increase in the fraction of cones in the *rd7* (from 1.65% of total retinal cells in WT to 3.2% in *rd7* [17]) would not be enough to account for such a shift since the magnitude of this increase in the fraction of higher oligomers we see here (∼2 fold increase from WT to *rd7*) is similar to what we see in the *Nrl^−/−^* wherein 100% of rods are converted to cone-like cells. This suggests that the hybrid rod cells [5], [17] of the *rd7* are likely the primary source of the changes seen in the RDS complexes. We have previously hypothesized that the differential role of RDS in rods vs. cones (and differential complex formation in rods vs. cones) is tied to RDS having different unknown binding partners in the two cell types. Indirect support for this hypothesis is provided here: hybrid rods of the *rd7* abnormally express many cone genes [5], [17] and also exhibit cone-like patterns of RDS complex formation. Future experiments may specifically address the existence of cone-specific RDS interacting partners to more directly assess this hypothesis.

Several pieces of our data suggest that the degeneration in the *rd7* is not related to a deficit in functional RDS. We observe decreases in OS protein levels, including RDS, even though we show that RDS mRNA levels are corrected in the NMP/*rd7*. Furthermore, neither the *rd7* nor the NMP/*rd7* retina exhibits major defects in RDS complex formation or mislocalization of RDS. More importantly, the ultrastructure of *rd7* and NMP/*rd7* OSs is very different from that seen in cases of RDS haploinsufficiency [27]. In cases where RDS deficiency underlies a rod defect, OSs have a characteristic whorl appearance [27]. We do not see this phenotype in the *rd7* or the NMP/*rd7*, both of which exhibit nicely flattened and organized discs, although OSs are much shorter than WT. These observations suggest that the reported similarities between the *rd7* and models of RDS haploinsufficiency [39] do not persist on the ultrastructural level.

It is clear, however, that expression of the NMP transgene exacerbates the rod defect in the *rd7*. We observe reduction in rod function and shortening of rod OSs in the NMP/*rd7* compared to *rd7* and WT. Likely as a consequence of OS shortening we also observe declines in the levels of proteins expressed in rods, including RDS and ROM-1. These defects do not appear to be a consequence of altering retinal transcription per se, as we observe no functional defect in NMP/*Nrl^−/−^* mice. *Nrl^−/−^* are affected by the lack of RDS [30], [37] and by RDS mutations [29], so the lack of a defect in NMP/*Nrl^−/−^* is not because the photoreceptors in the *Nrl^−/−^* retina are immune to alterations in RDS expression. Thus the question arises, why is there such a severe defect in the NMP/*rd7* retina while the NMP/*Nrl^−/−^* and the NMP/WT retinas exhibit no defects?

The most likely explanation is tied to the effects of NRL ablation vs. NR2E3 ablation. In the absence of NRL in mice, no rods are formed at all [2], yielding a stable population of cells which express cone genes and not rod genes. Normally, NRL activates NR2E3 in newborn photoreceptors that are committed to a rod fate. In a small population of early-born rods, absence of NR2E3 leads to the formation of the extra cones seen in the *rd7* [18]. In contrast, in a separate population of rod precursors (late-born rods [18]), the lack of NR2E3 expression results not in formation of additional cones but in formation of hybrid photoreceptors which express both rod and cone genes [5], [17], [18]. These hybrid rods are likely under stress due to the expression of abnormal cone genes and we hypothesize that inducing them to express extra RDS on top of the other abnormalities increases cellular stress leading to the exacerbated rod phenotype we observe in the NMP/*rd7*. This hypothesis is supported by the observation that the cones in the *rd7* are not in this hybrid state of expressing both rod and cone genes, and thus do not exhibit any defect when they are induced to express NMP. In conclusion, these data suggest that the defect in the *rd7* is not due to RDS haploinsufficiency and that expression of the NMP transgene may accelerate the degeneration of hybrid photoreceptors which abnormally express both rod and cone genes.

## Materials and Methods

### Ethics statement and animal care and use

All experiments and animal maintenance were approved by the local Institutional Animal Care and Use Committee (IACUC; University of Oklahoma Health Sciences Center, Oklahoma City, OK, U.S.A.) and conformed to the guidelines on the care and use of animals adopted by the Association for Research in Vision and Ophthalmology (Rockville, MD). The NMP/*rd7* transgenic mice were generated by cross-breeding our NMP transgenic mice [24] with *rd7* mice [15], [16] purchased from Jackson laboratories (Bar Harbor, ME). Non-transgenic wild-type (WT) littermates and *rd7* mice were used as controls. Animals were maintained in cyclic light (12 hours light, 12 hours dark, ∼30 lux).

### Antibodies

Primary antibodies were used as described in each section. Several antibodies used here were generated in-house and characterized previously [26], [40] including: 1) RDS-CT rabbit polyclonal (recognizing both endogenous murine RDS and transgenic NMP), 2) rabbit polyclonal S-opsin, and 3) rabbit polyclonal ROM1-CT. These were used at 1∶1000 for western blot (WB) and immunohistochemistry (IHC). mAB 3B6 recognizing transgenic RDS only (NMP, 1∶50 for IHC), and mAB 1D4 recognizing rhodopsin (1∶1000 on WB and IHC) were generously shared by Dr. Robert Molday (University of British Colombia, Vancouver, Canada). Rabbit polyclonal S-opsin (1∶10 on immunogold EM) and M-opsin (1∶30,000 on IHC, 1∶15,000 on WB) antibodies generously shared by Dr. Cheryl Craft (University of Southern California, Los Angeles, CA). Rabbit polyclonal anti rod-opsin (immunogold/EM 1∶10) was generously shared by Dr. Steven Fliesler (State University of New York, Buffalo, NY).

### Histology, transmission electron microscopy, and immunogold cytochemistry

The methods used for tissue collection, processing, plastic-embedding, and immunogold labeling were as described previously [30], [31], [37]. For light microscopy, 0.75 µm sections were observed and photographed with an Olympus BH-2 photomicroscope with a Nikon digital camera system. Thin (600–800 Å) sections for TEM were collected on copper 75/300 mesh grids and stained with 2% (w/v) uranyl acetate and Reynolds' lead citrate. Thin sections for immunogold were collected on nickel 75/300 mesh grids. Primary antibodies were used as described above, and secondary antibodies (1∶50) were AuroProbe 10 nm gold-conjugated goat anti-rabbit IgG; (GE/Amersham, Piscataway, NJ). Sections were viewed with a JEOL 100CX electron microscope at an accelerating voltage of 60 kV. OS length was measured using Adobe Photoshop CS5. OSs were measured in 3 central retinal fields/eye from plastic embedded sections and 3 eyes/genotype.

### Immunofluorescence Labeling

Eyes were harvested, dissected, fixed and embedded as previously described for paraffin sectioning (6 µm) [31], or cryosectioning (10 µm) [41]. Immunostaining was performed as described previously [29], [31] using the primary antibodies described above. Anti-mouse or anti-rabbit AlexaFluor 488 or 555 conjugated secondary antibodies (Life Technologies, Grand Island, NY) were used at a dilution of 1∶1000 for 1 hr at room temperature. Images were captured an Olympus BX-62 microscope equipped with a spinning disc confocal unit using a 40× (air, 0.9 NA) objective. Images were stored and deconvolved (no neighbors paradigm) using Slidebook® version 4.2.0.3. All images shown are single planes. For rosette quantification, transverse (superior-inferior) retinal sections containing the optic nerve head were examined (at least three sections per eye, and three eyes per genotype) and rosettes were counted across the whole section.

### qRT-PCR

Total RNA from frozen eyes was extracted using TRIzol (Life Technologies) as described previously [30]. RNase-free DNase treatment was performed with DNase (Life Technologies) to remove genomic DNA. cDNA synthesis by reverse transcription was performed and 2 µg of cDNA from each sample was used for qPCR. qRT-PCR was performed in triplicate on each cDNA sample with a MyIQ qRT-PCR machine (Bio-Rad) and cT values were calculated against the housekeeping gene beta-actin [42]. Melt curve analysis and agarose gel electrophoresis were performed to ensure that the PCR products were specific and of appropriate size. Primer sequences are found in **Table S1**.

### Electroretinography

ERGs were performed as previously described [26], [30]. Briefly, following overnight dark-adaption mice were anesthetized, and eyes were dilated. Electrophysiological function was assessed using the UTAS system (LKC, Gaithersburg, MD, USA). Assessment of rod photoreceptor function (scotopic ERG) was performed with a strobe flash stimulus of 157 cd-s/m^2^ intensity presented to the dark-adapted dilated eyes in a BigShot ganzfeld. To evaluate photopic response, animals were light adapted for 5 minutes under a light source of 29.03 cd/m^2^ intensity in a BigShot ganzfeld. Cone photoreceptor function (photopic ERG) was assessed by averaging responses to 25 strobe flash stimuli at either 530 nm (12.5 cd-s/m^2^, BigShot green LED light source), 365 nm (0.79 cd-s/m^2^), or white light (79 cd-s/m^2^, BigShot UV LED light source). At least 8 mice per genotype were analyzed.

### Western blot analysis and velocity sedimentation

Western blot and velocity sedimentation were performed as described previously [29], [31]. Briefly, retinas were homogenized on ice in solubilization buffer containing 50 mM Tris-HCl, pH 7.5, 100 mM NaCl, 5 mM EDTA, 1% Triton X-100, 0.05% SDS, 2.5% glycerol, and 1.0 mM phenylmethylsulphonyl fluoride (PMSF). Non-reducing velocity sedimentation was performed using continuous density gradients of 5–20% sucrose and 200 µg protein/sample. Molecular weight markers for the determination of tetrameric, octomeric, and oligomeric fractions were used and have been previously published [36], [43]. Summary graphs for velocity sedimentation experiments were prepared by summing the percent of total RDS found in each fraction. Experiments were repeated 3–6 times.

### Statistical analysis

Differences between genotypes were assessed by 1-way ANOVA with Bonferroni's post-hoc pairwise comparisons, or two-tailed Student's t-test (when only two groups were tested). P<0.05 was considered significant. Graphs are presented as mean ± SEM.

## Supporting Information

Table S1 Primer sequences used for qRT-PCR.
(PDF)
