# Supplementary material for: Overexpression of Retinal Degeneration Slow (RDS) Protein Adversely Affects Rods in the rd7 Model of Enhanced S-Cone Syndrome
Source: PLoS One. 2013 May 1;8(5):e63321. doi: 10.1371/journal.pone.0063321 (PMC3641132; doi:10.1371/journal.pone.0063321)
Supplement: Table S1 — Primer sequences used for qRT-PCR. (PDF) [file pone.0063321.s001.pdf]

SUPPORTING INFORMATION

**Gene**	**Sequence**
Total RDS F	ACCCCGAGTGTGAGAGTGAG
Total RDS R	AAAGGGGGTATCCACAGTCC
Transgenic RDS (NMP) F	GGAACTGATGAATGGGAGCA
Transgenic RDS (NMP) R	GGAAAGTCCTTGGGGTCTTC
Actin F	TGTTACCAACTGGGACGACA
Actin R	CTTTTCACGGTTGGCCTTAG

Table S1: Primer sequences used for qRT-PCR.
